# Brain Structures Associated with Internet Addiction Tendency in Adolescent Online Game Players

**DOI:** 10.3389/fpsyt.2018.00067

**Published:** 2018-03-06

**Authors:** Nannan Pan, Yongxin Yang, Xin Du, Xin Qi, Guijin Du, Yang Zhang, Xiaodong Li, Quan Zhang

**Affiliations:** ^1^Department of Radiology and Tianjin Key Laboratory of Functional Imaging, Tianjin Medical University General Hospital, Tianjin, China; ^2^Department of Psychology, Linyi Fourth People’s Hospital, Linyi, China; ^3^Department of Radiology, Linyi People’s Hospital, Linyi, China

**Keywords:** Internet gaming addiction, gray-matter volume, Internet addiction test, online game, adolescent

## Abstract

With the development of the Internet, an increasing number of adolescents play online game excessively, which leads to adverse effects on individuals and society. Previous studies have demonstrated altered gray-matter volume (GMV) in individuals with Internet gaming disorder (IGD), but the relationship between the tendency to IGD and the GMV across whole brain is still unclear in adolescents. In the present study, anatomical imaging with high resolution was performed on 67 male adolescents who played online game; and Young’s Internet addiction test (IAT) was conducted to test the tendency to IGD. FMRIB Software Library (FSL) was used to calculate the voxel-based correlations between the GMV and the IAT score after controlling for the age and years of education. The GMVs of the bilateral postcentral gyri (postCG), the bilateral precentral gyri (preCG), the right precuneus, the left posterior midcingulate cortex (pMCC), the left inferior parietal lobe (IPL), and the right middle frontal gyrus (MFG) were negatively correlated with the IAT score. The correlation still existed between the IAT score and the GMVs of the bilateral postCG, the left preCG, the left pMCC, and the right MFG after controlling for the total time of playing online game. When the participants were divided into two groups according to the IAT score, the GMVs of these IAT-related brain regions were lower in high IAT score subgroup (IAT score >50) than in low IAT score subgroup (IAT score ≤50). Our results suggested that the GMVs of brain regions involved in sensorimotor process and cognitive control were associated with the IGD tendency. These findings may lead to new targets for preventing and treating the IGD.

## Introduction

In the past decades, the Internet played an important role in our life. However, more and more adolescents surf the Internet and play online game excessively, which result in adverse effects on adolescents themselves and society. An epidemiological study demonstrated that Internet gaming disorder (IGD), a subtype of Internet addiction (IA) (1), was a very common mental health problem among Chinese adolescents (2). Therefore, more and more studies focused on the neuromechanism of IGD and aimed to contribute to the prevention and treatment of IGD.

Structural neuroimaging of brain could be used to investigate brain mechanisms about individual personality traits (3–5). Previous structural studies have found that individuals with IGD had structural abnormalities in gray matter (GM), such as decreased gray-matter volume (GMV) or GM density in multiple cortical and subcortical areas (6–11), and increased GMV in frontal and temporal regions (8, 12). These studies suggested that multiple brain areas in the frontal, temporal, parietal, and subcortical regions such as ventral striatum were associated with IA, which contributed to the understanding of the neuromechanisms of IA. However, the majority of previous studies just focused on the IA or IGD diagnosed by clinical questionnaire such as Internet addiction test (IAT), and compared the differences in behavior and brain function and structure between the IGD individuals and healthy controls. As a matter of fact, not all the individuals who play online game suffer from the IGD (13). Therefore, investigation of the structural correlations in online game players with different levels of tendency to IGD, not only the individuals with IGD diagnosis, is necessary.

Recently, three studies directly focused on the neural associations of the tendency to the IA. Wen and Hsieh (14) explored the relationship between the whole brain functional connections and the level of IA in a group of young adults (19–29 years) and found two networks mainly consisted of frontal regions were correlated to the tendency of IA. Li et al. (15) reported that the structure and functional connectivity of the right dorsolateral prefrontal cortex were positively correlated with the IAT score in a group of healthy young adults (18–27 years). A study by Kühn (16) revealed that the GMV of the brain regions within fronto-striatal network correlated to excessive Internet use assessed by IAT score. Additionally, previous studies have also demonstrated that the GMV changes were related to the online game addiction severity in the IGD subjects. For example, a study by Weng et al. demonstrated that the GMVs of the right orbitofrontal cortex and bilateral insula were positively correlated with the online game addiction severity in the IGD subjects (7). Cai et al. reported increased GMV of nucleus accumbens was associated with the IAT score in the IGD individuals (17). A study by Zhou et al. showed that lower GMV in the right orbitofrontal cortex was related to higher online video gaming addiction severity within the Internet gamers (18). These studies demonstrated that brain structures and functions were associated with the level of IA. However, the relationship between the tendency to IGD and the GMV across whole brain was not yet clearly evaluated in adolescents (14–18 years). The adolescent between 14 and 18 years of age is in a critical period of psychological development and is prone to addiction and adverse effects (19, 20). Many studies regarding the substance addiction paid close attention to adolescents aged from 14 to 18 years (21, 22). A large-sample study demonstrated that the IGD is very common in Chinese elementary and middle school students with a incidence of 22.5% among those students who play online games (2). Therefore, it is more necessary to investigate the brain structural correlations with the tendency to IGD in adolescents (14–18 years).

Furthermore, previous studies demonstrated that long-term online game playing could lead to structural reorganization of the brain in online game players (12, 23, 24). The GMVs in the ventrolateral prefrontal cortex, the dorsolateral prefrontal cortex, the supplementary motor area, and the rostral anterior cingulate cortex were correlated with the duration of online game playing in the adolescents with IA disorder (6, 25). Therefore, whether the duration of online game playing affects the relation between the GMV and the tendency to IGD is worth studying.

In the present study, 67 male adolescents (14–18 years) who played online games were recruited. The voxel-based correlation analysis was conducted to detect the brain regions associated with IAT score before and after controlling for the total time of playing online game. Based on the previous studies, the prefrontal-striatal circuits are closely related to the addiction. Ventral striatum participated in the habit learning and rewarding process involved in addiction (26, 27), and the reduced control effect of prefrontal cortex on rewarding process is one of the mechanisms of addiction (28, 29). Therefore, we hypothesized that the IGD tendency may be associated with the brain regions related to the cognitive control (prefrontal cortex) and the rewarding process (ventral striatum). This study may lead to new targets for preventing and treating the IGD in adolescents.

## Materials and Methods

### Subjects

Sixty seven right-handed adolescents (14–18 years old, average 15.54 ± 0.14) who played online game were recruited in this study. Twenty of 67 participants were the students of a Health School and 47 of 67 participants were the adolescents whose parents took them to a psychiatrist because of possible IGD. All participants received education for 6–12 years, ranging from primary school to senior high school. All of the participants spent more than 80% of the online time on playing online game. Only male adolescents were enrolled in this study because relatively small number of females play online games and suffer from IGD (2, 30). Exclusion criteria included the following: alcohol abuse or drug dependence; existence of any neurologic or psychiatric disease such as insomnia, migraines, tinnitus, and attention deficit hyperactive disorder; history of physical illness such as brain trauma, brain tumor, or epilepsy assessed according to clinical evaluations and medical records; MRI contradiction; and visible abnormalities on conventional MRI. The present study was approved by the Ethical Committee of Tianjin Medical University General Hospital, and all of the participants and their guardians provided written informed consent according to institutional guidelines.

### Questionnaire

Internet addiction test was used to assess the severity of the tendency to IGD in this study. The IAT consists of 20 items and the answers of these questions were described as 1–5 score (1 = “rarely” to 5 = “always”) (31). The total score of 20 items measures the severity of Internet dependency. The experience of online game playing was assessed *via* a self-report questionnaire that questioned about the length and amount of playing. The total time of playing online game was calculated as hours per day multiplied by the days of playing online games. Intelligence Quotient (IQ) of all participants was tested using Standard Raven’s Progressive Matrices. The anxiety and depression were texted by using the self-rating anxiety scale (SAS) and the self-rating depression scale (SDS).

### Structural MRI

Structural images were obtained using a Siemens 3.0 T scanner (Magnetom Verio, Siemens, Erlangen, Germany). A series of 192 contiguous sagittal high-resolution anatomical images were obtained using a three-dimensional T1-weighted volumetric magnetization-prepared rapid gradient-echo sequence with the following parameters: TR = 2000 ms, TE = 2.34 ms, TI = 900 ms, flip angle = 9°, FOV = 256 mm × 256 mm, slice thickness = 1 mm, matrix size = 256 × 256.

### Voxel-Based Morphometry (VBM) Analysis

All structural images were preprocessed with the VBM8 toolbox^1^ of the SPM8 (Wellcome Department of Imaging Neuroscience, London, UK)^2^ running on MATLAB R2010a (Math Works Inc., Sherborn, MA, USA). Three-dimensional geometric correction was performed during reconstructing the images. After that, the individual native images of all participants were segmented into GM, white matter (WM), and cerebral spinal fluid (CSF), and the GM segments were normalized to the Montreal Neurological Institute template by diffeomorphic anatomical registration through exponentiated lie algebra (DARTEL) (32). The registered GM images were then modulated by dividing the Jacobian of the warp field to correct for local expansion or contraction. The isotropic Gaussian kernel of 8-mm full width at half maximum was adopted to smooth the modulated GM images. The mean image of normalized GM from all participants was used to create a GM mask whose threshold was set at a value of 0.3 (pixels with computed GM fraction values >30% were selected). Then the GM mask was used as an explicit mask for the statistical analysis to exclude the pixels with low GM probability values.

### Statistical Analysis

Voxel-wise multiple regression analysis was carried out to explore the correlation between the GMV and the IAT score across all participants after controlling for the age and years of education. The non-parametric permutation approach (33) was accomplished by the randomize tool commanded in FMRIB Software Library (FSL)^3^. The threshold-free cluster enhancement (TFCE) analysis was performed as it combines cluster extent and height into one statistic and does not require an arbitrary choice of a cluster forming threshold (34). The correlation between the GMV and the IAT score was assessed using permutation-based non-parametric testing with 5,000 random permutations. The statistical threshold for significance was defined at *P* < 0.01. For clarifying whether the duration of online game playing affected the correlation between the GMV and the IAT, Voxel-wise multiple regression analysis was conducted again when adding the total time of playing online game as a nuisance covariate.

Clusters with correlation between the GMV and the IAT score were defined as regions of interest (ROIs), and the average GMV within each ROI was extracted. ROI-based correlation analysis was conducted between the average GMV and the IAT score after controlling for the age and years of education. Then, all of the participants were divided into two subgroups, the high IAT score group (IAT score >50, *N* = 30) and the low IAT score group (IAT score ≤50, *N* = 37). The difference in the GMV between the two subgroups was tested by General Linear Model analysis, controlling for the age and years of education. The significance levels were both set at *P* < 0.05.

## Results

Participants had a median score of 46 on the IAT which was used to assess the IGD tendency. Subjects spent average 5.5 h/day on playing online games and lasted for average 56 months. The clinical and demographic characteristics are listed in Table 1.

**Table 1:** Participant’s characteristics.

Item	Mean ± SD/median (range)
Age (years)	15.54 ± 0.14
Education (years)	9.40 ± 0.18
IQ	47.89 ± 0.76
Time of playing online game per day (hours)	5.47 ± 4.72
Duration of playing online game (month)	55.97 ± 31.71
Total time of playing online game (hours)	5760 (240–37,260)^a^
IAT score	46 (22–92)^a^
SAS	39.64 ± 7.61
SDS	44.81 ± 10.28

*IAT, Internet addiction test; IQ, intelligence quotient; SAS, self-rating anxiety scale; SDS, self-rating depression scale*.

*^a^The variables present with non-normal distribution*.

**Figure 1:**
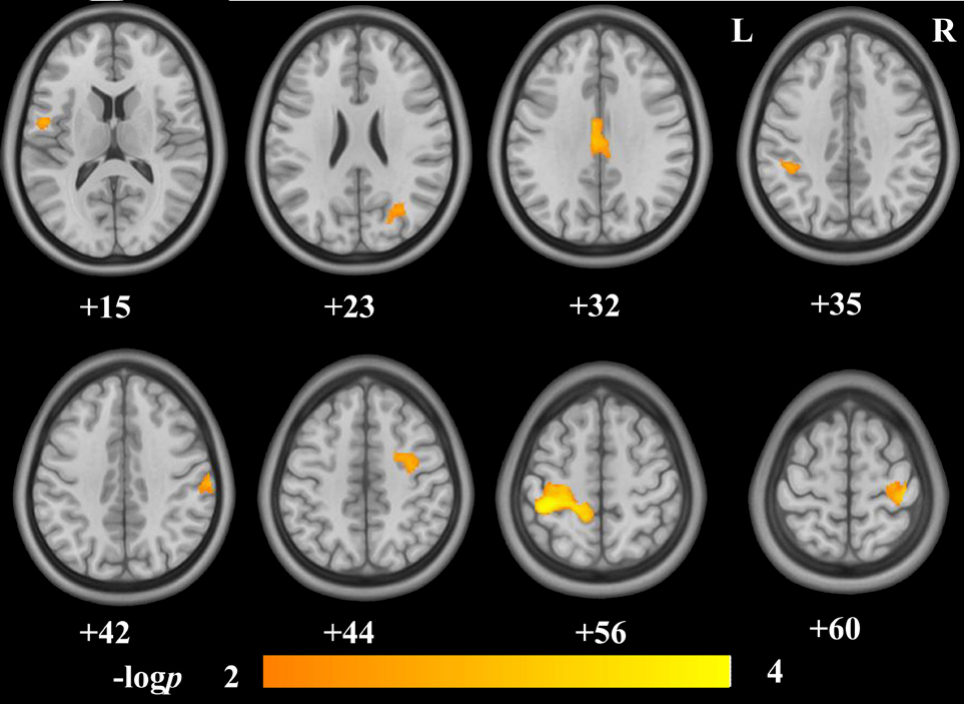
Brain regions showing negative structural correlates to Internet addiction test (IAT) score in adolescent online game players. The IAT score was negatively correlated to the gray-matter volumes (GMVs) of the bilateral postcentral gyri, the bilateral precentral gyri, the right precuneus, the left posterior mid cingulate cortex, the left inferior parietal lobule, and the right middle frontal gyrus. The numbers below the images are the Montreal Neurological Institute coordinates at *z*-axis. The colorbar represents the −log *p*.

**Figure 2:**
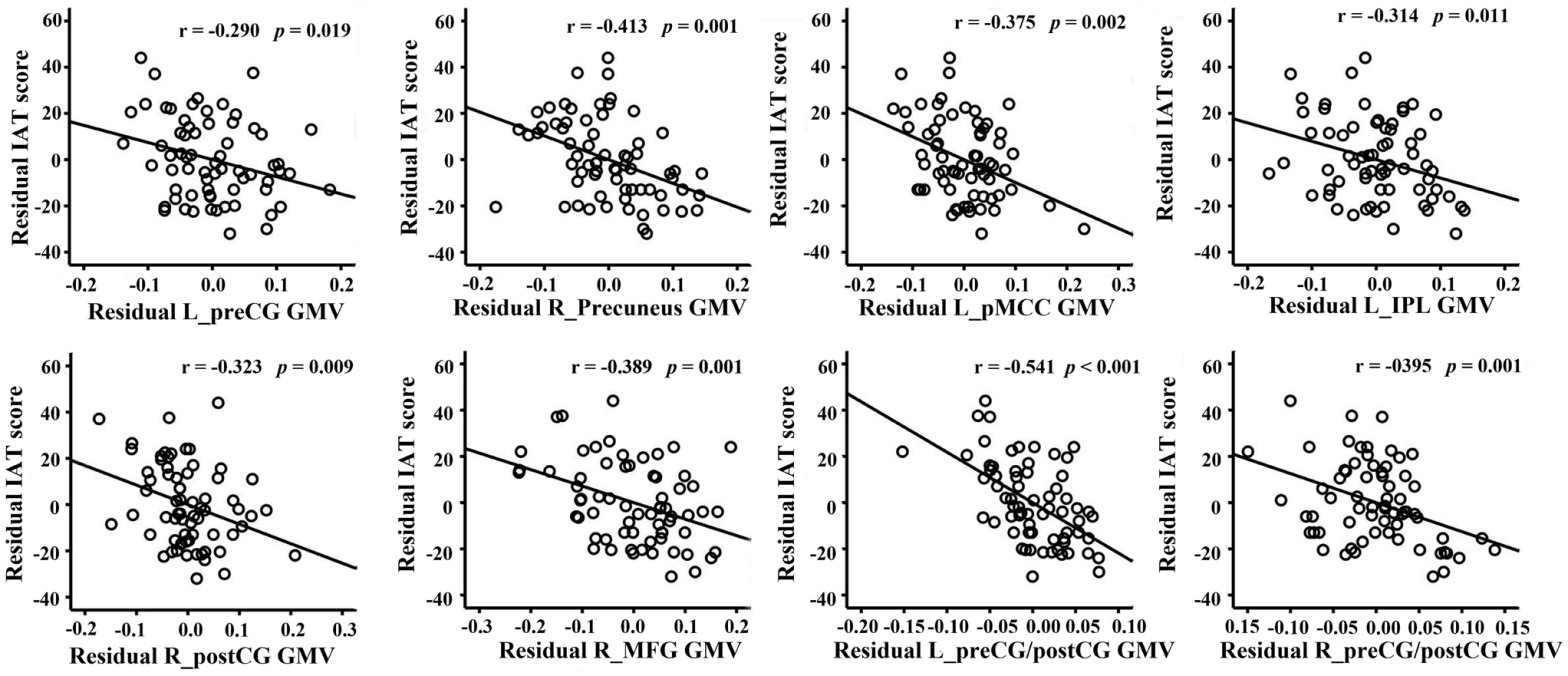
Regions-of-interest (ROI)-based correlation analysis between the gray-matter volume (GMV) and the Internet addiction test (IAT) score. The residual was used because the age and years of education were controlled during correlation analysis.

**Figure 3:**
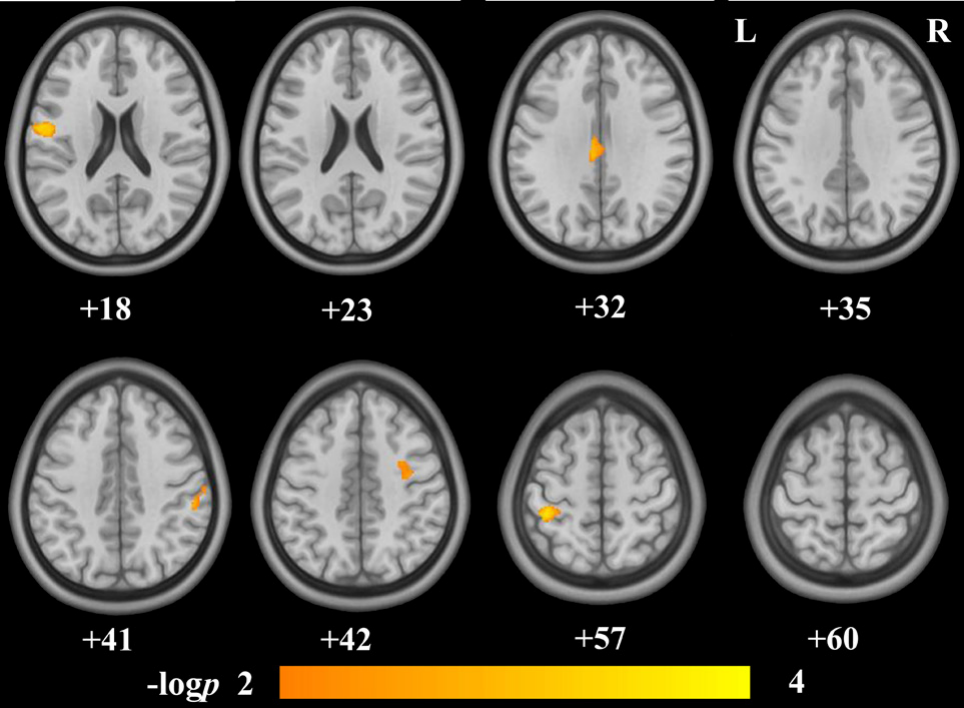
Brain regions showing negative structural correlates to Internet addiction test (IAT) score in adolescent online game players after controlling for the total time of playing online game. The IAT score was negatively correlated to the gray-matter volumes (GMVs) of the bilateral postcentral gyri, the left precentral gyrus, the left posterior mid cingulate cortex, and the right middle frontal gyrus. The numbers below the images are the Montreal Neurological Institute coordinates at *z*-axis. The colorbar represents the −log *p*.

Voxel-wise correlation analysis revealed that the GMVs of the bilateral postcentral gyri (postCG), the bilateral precentral gyri (preCG), the right precuneus, the left posterior midcingulate cortex (pMCC), the left inferior parietal lobe (IPL), and the right middle frontal gyrus (MFG) were significantly correlated to the IAT score (Figure 1; Table 2). Figure 2 shows the ROI-based correlations between the GMV and the IAT score. After the total time of playing online game was added as a nuisance covariate, the correlation still existed between the IAT and the GMV of the bilateral postCG, the left preCG, the left pMCC, and the right MFG (Figure 3; Table 3).

**Table 2:** Brain regions showed structural correlates to Internet addiction test (IAT) score.

Region	Peak MNI coordinates			*P*-value	Cluster size (voxels)
	*X*	*Y*	*Z*		
L_PreCG	−51	−3	15	0.0055	302
R_PreCG/PostCG	42	−25.5	60	0.0026	619
L_PreCG/PostCG	−40.5	−37.5	55.5	0.0002	4898
R_PostCG	63	−21	42	0.0044	262
R_Precuneus	30	−67.5	22.5	0.0053	502
L_pMCC	−4.5	−18	31.5	0.0040	555
L_IPL	−39	−39	34.5	0.0047	192
R_MFG	28.5	−3	43.5	0.0053	475

*IPL, inferior parietal lobule; MFG, mid frontal gyrus; MNI, Montreal Neurological Institute; PreCG, precentral gyrus; PostCG, postcentral gyrus; pMCC, posterior mid-cingulate cortex; L, left; R, right*.

**Table 3:** Regions showed structural correlates to Internet addiction test (IAT) score after controlling for the total time of playing online game.

Region	Peak MNI coordinates			*P*-value	Cluster size (voxels)
	*X*	*Y*	*Z*		
L_PreCG	−49.5	−7.5	18	0.0032	1,116
L_ PreCG/PostCG	−40.5	−37.5	57	0.0020	284
R_PostCG	54	−30	40.5	0.0055	159
L_pMCC	−6	−24	31.5	0.0049	222
R_MFG	34.5	−7.5	42	0.0063	173

*MFG, mid frontal gyrus; MNI, Montreal Neurological Institute; PreCG, precentral gyrus; PostCG, postcentral gyrus; pMCC, posterior midcingulate cortex; L, left; R, right*.

As seen in Table 4, when the participants were divided into the two subgroups according to the IAT score, the subgroup with high IAT score (IAT score >50) had lower GMV in the seven of eight regions compared with the subgroup with low IAT score group (IAT score ≤50) (*P* < 0.05).

**Table 4:** Regions-of-interest (ROI)-based comparisons of the gray-matter volume (GMV) between the two subgroups.

ROIs	High IAT score subgroup (*N* = 30)	Low IAT score subgroup (*N* = 37)	*T*	*P*
L_PreCG	0.465 ± 0.071	0.487 ± 0.067	−1.285	0.203
R_PreCG/PostCG	0.433 ± 0.046	0.462 ± 0.059	−2.229	0.029
L_ PreCG/PostCG	0.464 ± 0.044	0.507 ± 0.033	−4.604	<0.001
R_PostCG	0.524 ± 0.058	0.566 ± 0.071	−2.62	0.011
R_Precuneus	0.457 ± 0.071	0.506 ± 0.067	−2.882	0.005
L_pMCC	0.614 ± 0.062	0.649 ± 0.067	−2.148	0.035
L_IPL	0.496 ± 0.069	0.546 ± 0.066	−3.015	0.004
R_MFG	0.544 ± 0.103	0.620 ± 0.074	−3.50	0.001

*IPL, inferior parietal lobule; MFG, mid frontal gyrus; PreCG, precentral gyrus; PostCG, postcentral gyrus; pMCC, posterior mid cingulate cortex; L, left; R, right*.

## Discussion

In the present study, the association between the GMV and IGD tendency was evaluated within the whole brain in adolescent online game players. After controlling for the effect of the total time of playing online game, the GMVs of the bilateral postCG, the left preCG, the left pMCC, and the right MFG were still negatively correlated to the IGD tendency. The adolescents with lower GMV in the brain regions related to sensorimotor process and cognitive control had higher IGD tendency.

It was consistent with the hypothesis that the GMV in MFG, as a part of prefrontal cortex involved in cognitive controls (35, 36), was negatively correlated with the IGD tendency. Structural and functional abnormalities were widely reported in individuals with IGD (37–40). For example, less activation in the prefrontal cortex was found in the IA (40). Previous studies demonstrated the lower GM density and GMV in the prefrontal cortex in the IGD individuals (37, 39). Smaller amplitude of low-frequency fluctuation within the right MFG was also revealed in the IGD individuals (41). Abnormal activation in the prefrontal cortex was also found in drug-addicted individuals such as the marijuana users and the abstinent cocaine abusers (42–44). Similar changes in functional connectivity of the prefrontal cortex were revealed in the individuals with alcohol dependence and the individuals with IGD (45, 46). These studies demonstrated that the structural or functional condition of prefrontal cortex was associated with the addiction. In this study, the GMV of the right MFG was negatively correlated to the IAT score, and was lower in the high IAT score subgroup than that in the low IAT score subgroup. Structural abnormality in the right MFG might lead to the impairment of cognitive control in online game players. As a result, the online game players could not control their problematic online game playing and exhibited a higher tendency to the IGD.

Incongruent with the hypothesis, we did not find the GMV of the ventral striatum correlating with the IAT score. The ventral striatum is a critical region related to the addiction, and usually presents abnormal activation in individuals with addiction (26, 27). In our study, we focused on adolescent online game players but not only the IGD individuals, which might be a possible explanation to the negative result of ventral striatum. However, this negative result should be verified in the future study with large sample size.

Unexpectedly, the preCG, postCG, and the pMCC involved in the sensorimotor process showed negative correlations with the IAT score. The preCG played a major role in the motor planning and conducting (47). Adolescence is a critical period of neural development, and is prone to be affected by the environmental factors. Previous studies demonstrated that the alcohol and drug use might change the GMV in the developing brain of adolescents (48). A study showed longer use of the methamphetamine was associated with the GMV reduction in the preCG (49). In our study, the GMV of preCG was lower in the high IAT score subgroup than that in the low IAT score subgroup. Considering prevention and suppression of the action is conceptually associated with the primary motor cortex (50), the decreased GMV of preCG might be related to the IGD tendency. The postCG consists of the primary sensory cortex and is involved in integrating sensory information (24). The negative correlation between the GMV of the postCG and the IAT score means the lower GMV of this region in individuals with higher IAT score. Abnormal function connectivity of the postCG was found in adolescents with IGD (51). The decreased GMV and cortical thickness of the postCG were also revealed, respectively, in heroin users (52) and adolescents with online gaming addiction (53). The impaired postCG may lead to abnormality in receiving, processing, and integrating body-relevant signals and may fail to guide ongoing behavior related to arousal, attention, stress, reward, and conditioning, and finally associated with the addiction (54). In this study, negative structural correlations to the IAT score were also found in the left pMCC. The pMCC exhibits extensive functional connectivity with brain regions involved in the sensorimotor network (55, 56) and has important role in processing sensorimotor integration and motor control (57). The sensorimotor areas not only control the basic aspects of movement but can also shape human behavior (58). The functional properties of sensorimotor network may be relevant for automatized/compulsive behaviors in addiction (59). Sensorimotor cortex impairments were also reported in individuals with cocaine addiction (60, 61) and alcohol ingestion (62). Taken together, the reduction of the GMVs within the preCG, postCG, and the pMCC might have association with the abnormalities of the sensorimotor network, and further associated with the IGD tendency.

In the present study, the negative correlations between the IAT score and the GMVs of the right preCG/postCG, the left IPL, and the right precuneus disappeared after controlling for the effect of the total time of playing online game. The preCG/postCG was involved in sensorimotor process (63); the IPL and the right precuneus were closely related to the visual and intentional processing (64–66). Gaming process requires players to pay full attention to the tiny change in the screen for a long time then injures their visual ability (65), which might have a relationship with the GMV reduction in the visual attention-related regions. Previous studies demonstrated decreased GMV of precuneus (8) and decreased cortical thickness of the IPL (53) in the individuals with online game addiction. Our results indicated that the GMV reduction in some brain regions related to the visual attention and sensorimotor process was influenced by the total time of playing online game, namely had a cumulative effect of playing online game.

Several limitations should be noted in our study. First, although some correlations were revealed between the brain GMV and IAT score, the causality cannot be clarified in this correlation analysis. The observed lower GMV in the adolescents with higher IAT score may be a result of excessive online game playing or a preexisting condition which is sensitive to IGD. Second, the IAT is a subjective questionnaire and more objective methods for evaluating the tendency to IGD are needed. Third, the total time of playing online games was just a probable measure and might be not accurate enough. Fourth, we could not rule out the effect of game genre on the results, which should be considered in the future study. Finally, only male adolescents were recruited in our study. Therefore, the present findings are restricted to male adolescent online game players.

## Conclusion

In this study, the structural correlation to the IGD tendency was investigated in a group of adolescent online game players. The GMV of brain regions related to the sensorimotor process and cognitive control were found to be associated with the IAT score. The lower GMV of the regions related to sensorimotor process and cognitive control might attribute to the high IGD tendency, which might lead to new targets for preventing and treating the IGD in adolescents.

## Ethics Statement

The present study was approved by the Ethical Committee of Tianjin Medical University General Hospital, and all of the participants and their guardians provided written informed consent according to institutional guidelines.

## Author Contributions

NP, YY, XL, and QZ designed research. XQ, XD, GD, YZ, and QZ performed research. YY was involved in the clinical assessment. NP, YZ, GD, and QZ analyzed data. NP, YZ, XL, YY, and QZ wrote the paper.

## Conflict of Interest Statement

The authors declare that the research was conducted in the absence of any commercial or financial relationships that could be construed as a potential conflict of interest.

## Footnotes

^1^http://dbm.neuro.uni-jena.de/vbm/.

^2^http://www.fil.ion.ucl.ac.uk/spm/software/spm8.

^3^http://www.fmrib.ox.ac.uk/fsl/index.html.
